# The Use of Continuous EEG Monitoring in Intensive Care Units in The Netherlands: A National Survey

**DOI:** 10.1007/s12028-018-0525-9

**Published:** 2018-03-27

**Authors:** Danny M. W. Hilkman, Walther N. K. A. van Mook, Werner H. Mess, Vivianne H. J. M. van Kranen-Mastenbroek

**Affiliations:** 1 Department of Clinical Neurophysiology, Maastricht University Medical Center + (MUMC +), PO-box 5800, 6202 Maastricht, The Netherlands; 2 Department of Intensive Care Medicine, Maastricht University Medical Center + (MUMC +), Maastricht, The Netherlands

**Keywords:** Continuous EEG monitoring, Non-convulsive seizures, Critical illness, Intensive care, Neuromonitoring

## Abstract

**Background:**

Currently, continuous electroencephalographic monitoring (cEEG) is the only available diagnostic tool for continuous monitoring of brain function in intensive care unit (ICU) patients. Yet, the exact relevance of routinely applied ICU cEEG remains unclear, and information on the implementation of cEEG, especially in Europe, is scarce. This study explores current practices of cEEG in adult Dutch ICU departments focusing on organizational and operational factors, development over time and factors perceived relevant for abstaining its use.

**Methods:**

A national survey on cEEG in adults among the neurology and adult intensive care departments of all Dutch hospitals (*n* = 82) was performed.

**Results:**

The overall institutional response rate was 78%. ICU cEEG is increasingly used in the Netherlands (in 37% of all hospitals in 2016 versus in 21% in 2008). Currently in 88% of university, 55% of teaching and 14% of general hospitals use ICU cEEG. Reasons for not performing cEEG are diverse, including perceived non-feasibility and lack of data on the effect of cEEG use on patient outcome. Mostly, ICU cEEG is used for non-convulsive seizures or status epilepticus and prognostication. However, cEEG is never or rarely used for monitoring cerebral ischemia and raised intracranial pressure in traumatic brain injury. Review and reporting practices differ considerably between hospitals. Nearly all hospitals perform non-continuous review of cEEG traces. Methods for moving toward continuous review of cEEG traces are available but infrequently used in practice.

**Conclusions:**

cEEG is increasingly used in Dutch ICUs. However, cEEG practices vastly differ between hospitals. Future research should focus on uniform cEEG practices including unambiguous EEG interpretation to facilitate collaborative research on cEEG, aiming to provide improved standard patient care and robust data on the impact of cEEG use on patient outcome.

## Introduction

Considerable interest exists on methods for monitoring of the brain in intensive care unit (ICU) patients, such as continuous electroencephalographic monitoring (cEEG). cEEG is the only contemporary available diagnostic tool for *continuous* monitoring of brain *function* offering notable advantages, closely related to the indications of its use, compared to the use of (repeated) routine EEG, other monitoring techniques or no monitoring at all [1, 2]. The most common indication for requesting cEEG is detection of non-convulsive seizures and status epilepticus. Other major indications are identification of cerebral ischemia, monitoring of sedation, assessment of severity of encephalopathy and of efficacy of therapy for seizures and status epilepticus and prognostication. [1, 3]. However, the exact relevance of routinely applied ICU cEEG remains unclear mainly due to ambiguous interpretation of certain EEG patterns and lack of standardized treatment protocols [2, 4, 5].

Despite ambiguity on interpretation of ICU cEEG, several international guidelines recommend its use. The European Society of Intensive Care Medicine (ESICM) recommends cEEG for refractory status epilepticus and suggests its use for diagnosing and managing status epilepticus and suspected ongoing seizures in comatose patients with unexplained persistent altered consciousness [6]. In a 2015 consensus statement, the American Clinical Neurophysiology Society (ACNS) recommends cEEG monitoring for diagnosis and management of non-convulsive seizures and status epilepticus, and other paroxysmal events [1]. Despite these recommendations, multiple factors seem to hamper its widespread clinical implementation [2]. However, as information on the actual implementation of cEEG, especially in Europe, is still scarcely available, identification and resolution of factors limiting its use remain difficult. Most of the information on contemporary cEEG practices available is derived from studies conducted in North America [7–9]. Concerning Europe, only two studies have been published: a German survey on scoring, monitoring, and parameter targeting in the ICU briefly mentioning cEEG [10] and a concise survey on the use of ICU cEEG in the Netherlands [11].

To gain more knowledge on the implementation of cEEG, this nationwide study explored the current practices of cEEG use in adult ICU departments in different types of hospitals in the Netherlands (as a representative example European country). This study focuses on organizational and operational factors influencing cEEG use. In addition, we explored how cEEG use has developed over time and which factors are considered relevant for abstaining from its use. Knowledge on contemporary cEEG practices may potentially guide research and development of guidelines on its implementation and use, thereby improving standard patient care.

## Methods

A national survey in Dutch language was carried out to obtain information on contemporary cEEG practices in adult ICU departments in the Netherlands.

The first survey was developed by a neurologist specialized in clinical neurophysiology/EEG. The survey was evaluated by two neurologists specialized in clinical neurophysiology, who assessed relevance of questions, technical issues, and flow of the survey resulting in minor revisions. For the second survey, this procedure was repeated with the addition of an intensivist as developer and two intensivists for evaluation of the survey. Ultimately, two surveys, one directed at neurologists and the other directed at intensivists, were developed.

The survey for neurologists consisted of 40 questions; 32 questions in multiple choice format and 8 questions in open format. The survey for intensivists consisted of 33 questions; 26 questions in multiple choice format and 7 questions in open-ended format.

Thirty questions, identical in both surveys, focused on general information of the respondents and the intensive care departments, as well as different aspects of cEEG. Topics included were: current and historical use of cEEG, organizational aspects, patient management, reviewing and reporting. The survey sent to neurologists included ten additional questions, focusing on specifics of review and reporting, like software, quantitative analyses, and EEG terminology used. The survey sent to intensivists included three additional questions focusing on the involvement of ICU staff. The final question in both surveys was an open-ended question format where respondents could add any further information they believed to be relevant.

A total of 82 neurology and 82 intensive care departments of all general (*n* = 48), teaching (*n* = 26) and university hospitals (*n* = 8) in the Netherlands were approached by e-mail and asked to complete one survey per department. In the Netherlands, a separate clinical neurophysiology specialty does not exist. Neurologists are trained to practice both neurology and clinical neurophysiology. In this study, merely for practical purposes, we refer to both using the term “neurologist.” Furthermore, in the Netherlands, all medical specialists are board-certified within their specialism and are obliged to re-certify every 5 years to stay professionally active.

The surveys were constructed and administered using Limesurvey (Version 2.05 + Build 140404), in 2014 and 2016, respectively.

Quantitative survey data were analyzed using Excel (Microsoft Excel for Mac version 15.03, build 170107) and SPSS (IBM SPSS Statistics version 22, build 22.0.0.0.). Descriptive statistics for comparing types of hospitals and specialists were performed using the Chi-square test, Fisher’s exact test, or Fisher’s Freeman-Halton test where applicable. Statistical significance was defined by a two-tailed *p* value < 0.05.

Qualitative data analysis was performed applying generally accepted principles of open, axial, and selective coding. Sentences containing information pertaining to separate subjects were coded separately. Coding was performed by the primary investigator (DH) and cross-checked by two co-investigators (WvM/VvK). Any discrepancies were discussed until consensus was reached.

## Results

### Response Characteristics

The response rate for neurology departments was 53% and for intensive care departments 58%, resulting in an institutional response rate of 78% (university hospitals 100%; teaching hospitals 77%; general hospitals: 75%). In 28 hospitals, both a neurologist and an intensivist responded (5 university hospitals, 10 teaching, and 13 general hospitals). For detailed results see Table 1.

**Table 1:** Characteristics of respondents and hospitals

Type of hospital (number of approached hospitals)	University hospital (*n* = 8)	Teaching hospital (*n* = 26)	General hospital (*n* = 48)
Respondents (response rate)	8 (100%)	20 (77%)	36 (75%)
Only neurologist	5	5	8
Only intensivist	0	5	15
Both neurologist and intensivist	3	10	13
Specialism			
Intensivist	2	13	28
Neurointensivist	3	2	0
Clinical neurophysiologist/neurologist	8	11	7
Neurologist	0	4	14
Size of ICU (number of beds)			
< 10	0	1	25
10–19	0	8	11
20–24	2	9	0
25–30	4	1	0
> 30	2	1	0
Number of hospitals with dedicated neurological ICU (number of beds)	2 (6–9)	1 (5)	0
Main type of patient admitted to ICU			
Mixed	8	17	30
Internal	0	1	2
Surgical	0	2	4
Number of neurological patients/month			
< 10	3	13	35
10–14	2	7	1
15–20	1	0	0
> 20	2	0	0

*ICU* intensive care unit

There are no statistically significant differences between responses from the neurologists and the intensivists. Consequently, unless stated otherwise, the presented results consist of the combined responses from both neurologists and intensivists. Furthermore, besides the size of the ICU departments (*p* = 0.001) and number of neurological patients treated (*p* = 0.048), there are no significant differences between the three types of hospitals. In the next sections, the results on different subtopics of the survey will be consecutively discussed

### Current cEEG Monitoring Use and Evolution Over Time

**Fig. 1:**
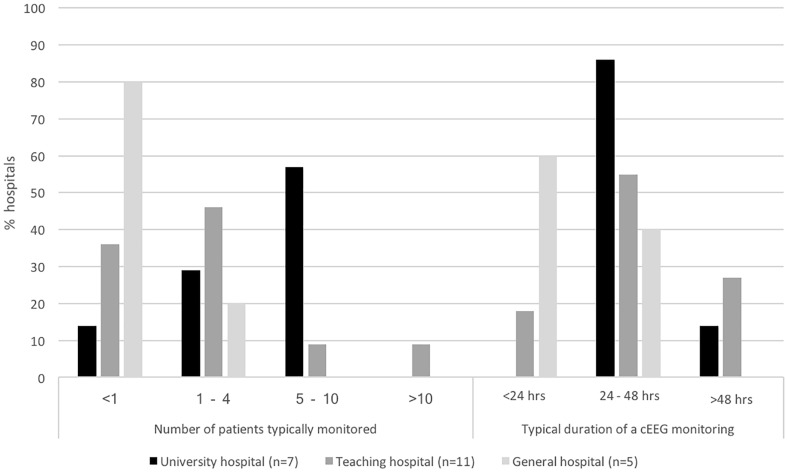
Average number of patients monitored per month and average duration of cEEG monitoring per hospital type

Over the last decade a growing number of hospitals used cEEG in the ICU in an overall growing number of patients. Currently, all but one university hospitals use cEEG on the ICU (*n* = 7, 88%), while teaching and general hospitals do so to a lesser extent (*n* = 11, 55% and *n* = 5, 14% resp.). In 2008, 13 hospitals performed cEEG on the ICU. Since then one university, six teaching and three general hospitals started using cEEG. Overall, 36% of Dutch hospitals used cEEG in 2016 versus 21% in 2008. In all but two hospitals the neurologist was involved in the decision to introduce cEEG in the ICU. If cEEG monitoring is used, its volume differs between hospital types. Results show that patients are monitored most frequently in university hospitals and least frequently in general hospitals (Fig. 1).

Of hospitals already performing cEEG in 2008, 10 currently perform more cEEG recordings than in 2008, two perform approximately the same number and one performs less recordings. Overall, university and teaching hospitals monitor a growing number of patients as opposed to 2008. In general hospitals, the extent of cEEG use is unaltered (*p* = 0.006).

Of respondents from hospitals currently *not* using cEEG (*n* = 41, 64%), 16 report never having considered using cEEG (39%), 7 assume cEEG to be useless (17%), 13 consider cEEG not feasible (32%), and 5 do not use cEEG for other reasons (12%). In the latter group, two general hospitals plan to start cEEG soon. If cEEG monitoring was deemed useless, the reasons indicated were: insufficient evidence for an effect on patient outcome (*n* = 2), too few eligible patients (*n* = 3), and no added value over not using cEEG (*n* = 2).

### Organizational Considerations

There are many similarities and some differences between hospitals in the way cEEG is organized. cEEG recordings can be started during office hours in all hospitals using cEEG (*n* = 23). To a lesser degree, recordings can be started in weekends (83%), in the evening hours (70%), and in the night (48%). In university and teaching hospitals, cEEG most often lasts at least 24–48 h, while in general hospitals, nearly all cEEG recordings last less that 24–48 h (Fig. 1).

Data on the use of ICU personnel for cEEG were available for 11 hospitals. In only three hospitals, ICU personnel provide a primary interpretation of the cEEG recording. In two hospitals, ICU personnel perform additional electrode maintenance separate from maintenance delivered by technicians.

In most hospitals performing cEEG (*n* = 16; 70%), a neurologist is available 24/7 for emergency review. If not, insufficient availability of personnel (*n* = 4) or insufficient technical resources, particularly tele-monitoring facilities (*n* = 5), were reported reasons. Overall, 57% of hospitals have tele-monitoring facilities, yet university, teaching and general hospitals differ markedly in its availability (100, 64, and 0% resp.; *p* = 0.013).

### Use in Patient Management

In all university and teaching hospitals, the neurologist, generally in collaboration with the intensivist, is involved in the decision on eligibility for cEEG monitoring. In general hospitals, the decision is typically made by the neurologist alone, rarely in collaboration with, or by the intensivist alone.

**Fig. 2:**
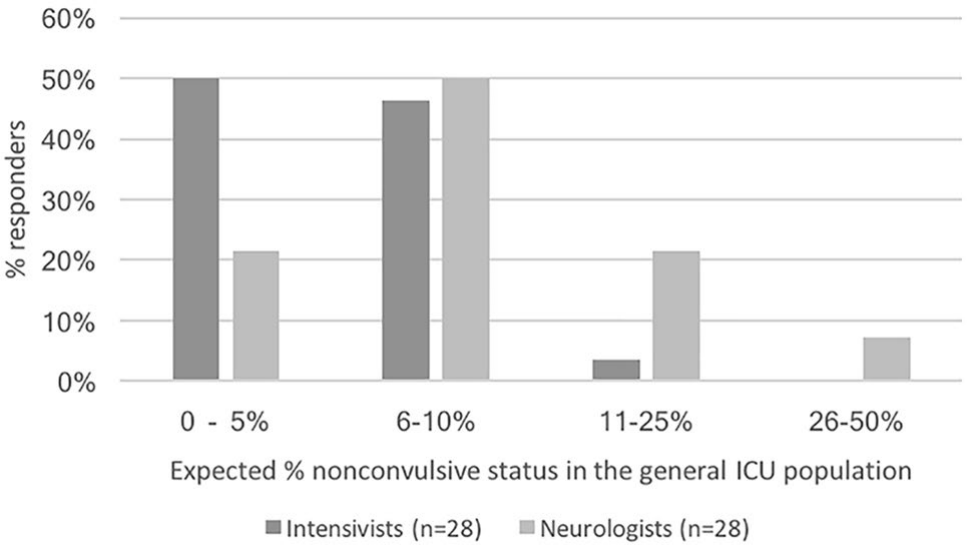
Percentage of non-convulsive status epilepticus in the general ICU population as expected by respondents. The differences between neurologists and intensivists do not reach statistical significance

**Fig. 3:**
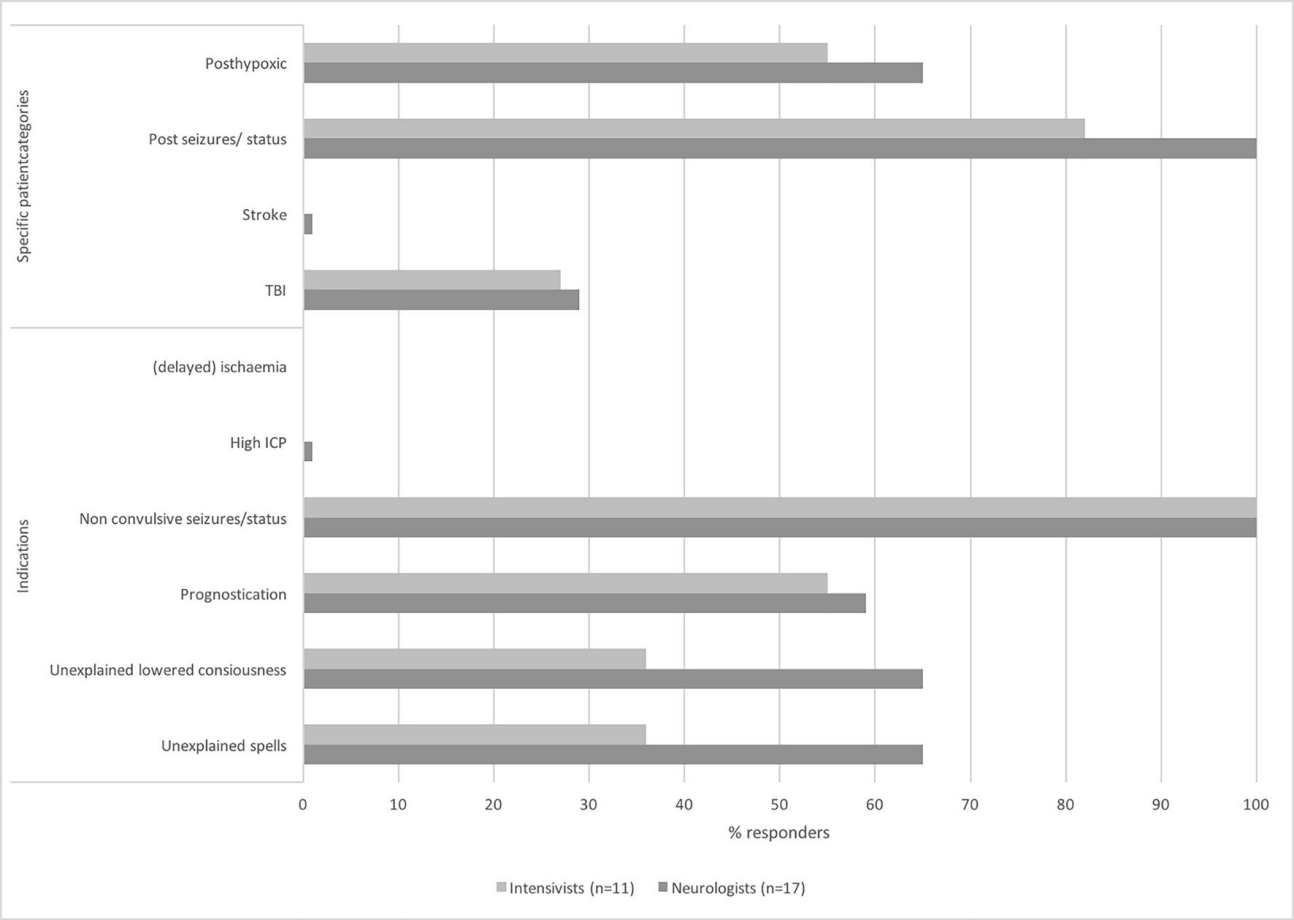
Monitored patient categories and cEEG indications as reported by neurologists and intensivists

As shown in Fig. 2, almost all intensivists and neurologists expect non-convulsive status in < 25% of the general ICU population. Additionally, in case of clinical suspicion of non-convulsive seizures or status epilepticus, all respondents use cEEG as displayed in Fig. 3. Furthermore, just over half of all respondents use cEEG for prognostication. Conversely, cEEG is never used for monitoring (delayed) cerebral ischemia and rarely for raised intracranial pressure in traumatic brain injury.

All respondents indicate that cEEG contributes to clinical decision making. cEEG results always contribute to clinical decision making in 8 hospitals. In the remaining 15 hospitals, cEEG results are perceived to contribute less frequently.

### Review and Reporting

**Fig. 4:**
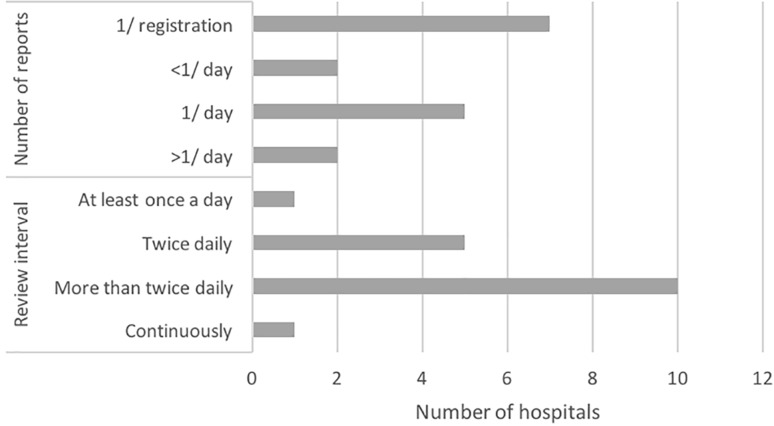
Number of reports written per cEEG recording and time intervals at which cEEG is reviewed

Review and reporting practices regarding cEEG differ substantially between hospitals. cEEG is reviewed at various intervals, and a varying number of reports is written per cEEG recording. As illustrated in Fig. 4, few hospitals use specific analyses supporting review. In only one hospital locally developed seizure detection software is used. Furthermore, in only six hospitals quantitative analyses are used. Reasons for refraining from using quantitative analyses are: considered to be of no added value (*n* = 3), no software available (*n* = 3), no expertise (*n* = 2), and not satisfied with the results (*n* = 2).

Finally, in almost half of the hospitals, the ACNS’s standardized critical care EEG terminology is used for review and reporting cEEG (53%), and in the others, no specific terminology is applied (47%).

## Discussion

The results of this nationwide survey exploring aspects of cEEG practices in different types of hospitals in the Netherlands indicate that ICU cEEG is used in nearly all Dutch university and most teaching hospitals, while its use in general hospitals is low. In addition, the study reveals that there is an increase in ICU cEEG use in the Netherlands similar to the USA [9]. In line with the findings of Ney et al. and Abend et al. in the USA [7, 8] this increase is mainly seen in university and teaching hospitals, probably explained by the fact that these are usually among the first to adopt new techniques for patient care and research. However, even in general hospitals, there is a modest increase in cEEG use even though these, usually smaller, hospitals treat comparably few cEEG eligible patients. In the Netherlands, on average, less than 10 ICU patients are monitored by cEEG each month, even in university hospitals. In contrast, in the USA, approximately 85% of medical centers monitor between 11 and 40 patients each month [9]. However, the average size of a (neurological) ICU in the USA is likely larger than in the Netherlands. Furthermore, the number of patients monitored relative to the size of the (neurological) ICU is unknown. Therefore, the difference in relative number of patients monitored in the Netherlands compared to the USA might be substantially lower.

Reasons for not performing cEEG are diverse. Most respondents indicate that cEEG implementation was never considered or was considered not feasible. A smaller group reports that cEEG was considered medically useless. A lack of definitive proof that patient outcome is influenced by cEEG use is one of the reasons why cEEG is considered less useful. Patient management decisions are highly diverse and interpretation of cEEG-findings ambiguous between hospitals and even individual physicians [2]. Also, proof of an effect of cEEG use on patient outcome may be difficult to establish since cEEG is a *diagnostic* tool. Possible effects of cEEG on patient outcome solely depend on patient management decisions *based on* cEEG findings. Nevertheless, currently two European multicenter randomized controlled trials are being performed aiming to relate cEEG use to patient outcome in specific patient populations. One is a Dutch study on treatment of patients with electrographic status epilepticus after cardiopulmonary resuscitation, aiming to assess whether its treatment in coma improves patient outcome (TELSTAR trial—clinical trials.gov NCT02056236) [12]. Furthermore, there is a Swiss study aiming to, among other things, to assess whether the use of cEEG in patients with impaired consciousness improves outcome (CERTA trial—clinical trials.gov NCT03129438).

Despite these limitations, all responding physicians using cEEG report that it contributes to some extent to clinical decision making. Reasons for the perceived lack of feasibility have not been specifically investigated; however, they might be related to a lack of expertise, shortage of 24/7 availability of specialized personnel, and the balance between potential patient benefit and labor intensity of cEEG. Possibilities of lowering labor intensity of cEEG could be implementation of specific detection algorithms, tele-monitoring, or outsourcing cEEG interpretation.

In 70% of hospitals, there is a 24/7 availability of the neurologists for (emergency) review of the traces. In general hospitals, the employment of too few specialized neurologists is identified as a reason for a less than 24/7 cEEG review availability. Also, within all types of hospitals, a lack of tele-monitoring capabilities is frequently reported as part of the reason for a less than 24/7 cEEG review availability even though comprehensive technical guidelines on cEEG including the use of tele-monitoring [13] and specifically on EEG tele-monitoring [14] have been published.

Indications prompting cEEG are largely comparable between the Netherlands and the USA [9]. Results show that the major indication prompting cEEG is detection of non-convulsive seizures/status epilepticus and monitoring of its treatment. Respondents are aware of the actual chance of presence of non-convulsive status epilepticus (NCSE) in the general ICU population. Nearly all respondents anticipate a < 25% chance, in agreement with several studies reporting percentages ranging from 5 to 21% [15–19]. The presence of NCSE is independently associated with higher mortality in the ICU population, while its treatment needs to be carefully fine-tuned considering the potential risks of treatment itself [20]. Detecting NCSE in comatose ICU patients is obviously challenging without the use of cEEG because of its relative or absolute lack of clinical symptoms. The introduction of cEEG in the ICU has substantially increased the detection rate of NCSE compared to using short-term EEG only [19]. In a 2013 consensus statement, the ECSIM strongly *recommends* the use of cEEG as a diagnostic modality for refractory status epilepticus. Conversely, it only *suggests* cEEG for patients with status epilepticus and suspected seizures [6]. The results of our study reveal that, in accordance with this consensus statement, all Dutch hospitals, if available, request cEEG for identification and management of non-convulsive seizures and status epilepticus. Prognostication in cardiac patients is the second largest reason for requesting cEEG in the Netherlands, in compliance with accumulating evidence in favor of using EEG for this purpose [2]. The ESICM suggests using EEG to improve prognostication in patients after cardiac arrest but does not explicitly suggest cEEG [6]. Studies have revealed that certain EEG patterns predict poor outcome [21–24] while cEEG can also assist in identifying patients with a favorable prognosis in selected cardiac patient populations [25–28]. The ESICM suggests to use cEEG to detect ischemia in comatose patients with subarachnoid hemorrhage (SAH) [6]. A few small studies show that detection of delayed cerebral ischemia using cEEG is feasible with high sensitivity in patients with SAH [29]. In the USA, only a minority of physicians use cEEG for detection of delayed cerebral ischemia [9], whereas in our survey, cEEG use for this purpose was not reported at all. Its explanatory reasons can only be speculated upon and may include a lack of knowledge on this indication for ICU cEEG.

Review practices differ substantially between hospitals. All but one review traces intermittently, yet at considerably different intervals. Hence, most hospitals provide rather a continuous EEG *recording* instead of a continuous EEG *monitoring,* making timely identification and treatment of non-convulsive seizures and status epilepticus less likely. However, longer duration of status epilepticus generally leads to more difficult treatment, possibly because of time-dependent pharmacoresistance [30, 31].

To solve this issue, there are several methods for moving toward continuous review of EEG traces, including using quantitative EEG trends, using specialized detection algorithms, and training ICU staff [2]. Quantitative analysis can be used as a first screening of cEEG traces for identification of regions of interest in the underlying raw EEG signal. As such, quantitative analysis guided review of cEEG can reduce the review time by almost 80%, compared to traditional review [32]. In the USA, 52% of physicians use quantitative analysis when reviewing ICU cEEG [9]. However, our respondents report that quantitative analysis is scarcely used and if so practices vary significantly. The reason for this difference between Dutch and American use of quantitative analysis is unclear. Furthermore, specialized detection algorithms can offer an automated first screening of raw EEG traces. However, except in one hospital, specialized detection algorithms in the Netherlands are not used. Lastly, evidence suggests that ICU staff can be trained to achieve a sufficient level of seizure identification using quantitative EEG trends [33]. Yet, only 3 of 11 hospitals involve ICU personnel in the initial review or maintenance of cEEG. In summary, although continuous review of EEG traces can be facilitated in several ways, none of these methods is frequently used in contemporary practice.

Another prominent finding in our study is that little over half of the respondents use uniform EEG terminology, i.e., the ACNS standardized critical care EEG terminology [34]. The influence of ambiguous EEG terminology on standard clinical practice is unknown. Yet, for research purposes and input for the development of guidelines unambiguous descriptions of EEG are invaluable. Recently, the revised European EEG terminology consensus statement, which incorporates the ACNS terminology, has been published: the “Standardised Computer-based Organised Reporting of EEG” (SCORE) [35]. SCORE may contribute to standardization of European cEEG reporting.

In contrast with the reported different practices between hospitals, no statistical differences between responses of neurologists and intensivists were found, suggesting alignment of policy and practice regarding cEEG use in the ICU resulting from a collaborative effort.

This study has some limitations. The number of closed-format questions predominated, restricting answering options. Yet, respondents had ample opportunity to provide narrative feedback in an open-ended question format. Also, the surveys were not conducted simultaneously, but approximately 2 years apart due to unanticipated logistical circumstances and changes in the research group. Though, there was no statistically significant difference between the responses from both surveys, this delay does introduce a possible bias since we do not know for certain if any cEEG-related changes have occurred in the responding medical centers within this time frame. Furthermore, a bias by more favorable responses regarding cEEG usage by a subset of experienced hospitals with special interest in this topic cannot be excluded. However, the relatively high response from approximately 3/4 of all Dutch hospitals may have limited this bias. Lastly, we acknowledge that the results may not be generalizable to other European countries due to differences in culture, organization of healthcare, legislation, or availability of expert personnel.

## Conclusion

cEEG is in the process of becoming a standard diagnostic modality in Dutch ICUs. However, cEEG practices still vastly differ between hospitals independent from their type. Furthermore, half of the Dutch teaching and general hospitals refrain from using ICU cEEG for different reasons, including perceived non-feasibility and lack of evidence on the effect of cEEG use on patient outcome.

Future research should focus on lowering cEEG workload while realizing real-time 24/7 cEEG *monitoring* as opposed to *recording only*. It should also concentrate on unambiguous EEG interpretation and patient management providing uniform cEEG practices. This will increase collaborative research possibilities regarding cEEG use, interpretation, and cEEG guided practices, supposedly providing robust data on the impact of cEEG use on patient outcome, in addition to improving standard patient care.
